# Lung microbiota: implications and interactions in chronic pulmonary diseases

**DOI:** 10.3389/fcimb.2024.1401448

**Published:** 2024-08-19

**Authors:** Jing Zhou, Wang Hou, Huilin Zhong, Dan Liu

**Affiliations:** Department of Pulmonary and Critical Care Medicine, West China Hospital, Sichuan University, Chengdu, Sichuan, China

**Keywords:** lung microbiota, chronic pulmonary diseases, microbial metabolomics, chronic obstructive pulmonary disease (COPD), microbial genomics

## Abstract

The lungs, as vital organs in the human body, continuously engage in gas exchange with the external environment. The lung microbiota, a critical component in maintaining internal homeostasis, significantly influences the onset and progression of diseases. Beneficial interactions between the host and its microbial community are essential for preserving the host’s health, whereas disease development is often linked to dysbiosis or alterations in the microbial community. Evidence has demonstrated that changes in lung microbiota contribute to the development of major chronic lung diseases, including chronic obstructive pulmonary disease (COPD), idiopathic pulmonary fibrosis (IPF), asthma, and lung cancer. However, in-depth mechanistic studies are constrained by the small scale of the lung microbiota and its susceptibility to environmental pollutants and other factors, leaving many questions unanswered. This review examines recent research on the lung microbiota and lung diseases, as well as methodological advancements in studying lung microbiota, summarizing the ways in which lung microbiota impacts lung diseases and introducing research methods for investigating lung microbiota.

## 1 Introduction

**Figure 1:**
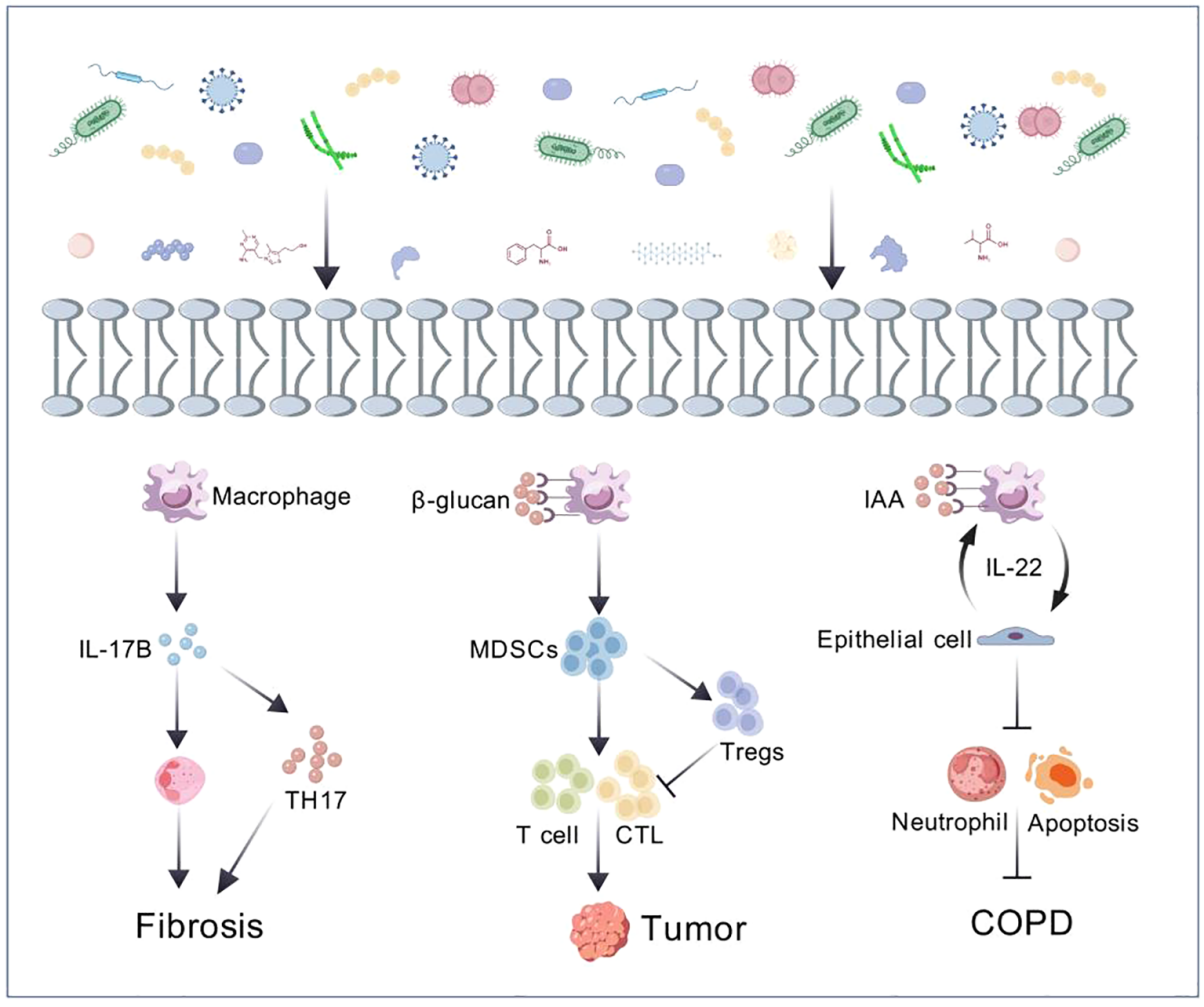
The crosstalk between microbiome and host (Yang et al., 2019; Liu et al., 2023; Yi et al., 2022).

The lung microbiota is an important component of the human microbiota (Kovaleva et al., 2019). It consists of the entire microbial community in the lungs, including bacteria, viruses, and fungi, forming a biological system that interacts with the host’s lung microenvironment in cellular signaling pathways and metabolic products, influencing each other. Studies have shown that the lung microbiota is established at birth, and the respiratory microbial community continues to develop during the first two years of life (Chu et al., 2017). The establishment of the microbiota is a crucial factor in the formation of a mature lung immune system and in protecting the lungs from harmful inflammatory responses (El Tekle and Garrett, 2023). It is involved in the normal development of the respiratory tract, regulating respiratory immunity, and maintaining respiratory health by preventing the spread of pathogens (Hou et al., 2022). Any dynamic system can be dysregulated by internal or external factors, and the same is true for lung microbiota. The imbalance in the lung microbiota may trigger or exacerbate respiratory diseases such as chronic obstructive pulmonary disease, asthma, and lung cancer (**Figure 1**) (Maddi et al., 2019). Investigating the mechanisms of lung microbiota and its microenvironment changes in the occurrence and development of lung diseases is of significant importance in exploring new potential therapeutic targets.

Although research on the lung microbiota is still in its early stages compared to the well-studied gut microbiota, studies have found that the microbial community in lung tissue also plays a functional role in the progression of lung diseases (Meng et al., 2023; Natalini et al., 2023a). Furthermore, on account of the unique characteristics of the lung microbiota (significantly smaller scale than the gut microbiota but highly diverse and more sensitive to environmental influences and greatly affected by oral and upper respiratory tract microbiota), the development of research techniques targeting the lung microbiota is essential for advancing research. To comprehensively understand the role of local microbiota in diseases, this review reports on the microbial characteristics of several chronic lung diseases, the mechanisms by which the microbiota promotes the occurrence and development of diseases, and recent cutting-edge research methodologies related to lung microbiota.

## 2 Microbiome characteristics of different chronic pulmonary diseases

### 2.1 Lung microbiome characteristics

The lung microbiota are mainly obtained from the exchange of nasopharynx, oropharynx and ambient air (Tsay et al., 2021). The lung microbiota of healthy individuals is mainly composed of *Streptococcus (Firmicutes), Fusobacterium (Fusobacteria), Haemophilus (Proteobacteria), Bacteroides (Bacteroidetes), Pseudomonas (Proteobacteria), Prevotella (Bacteroidetes)*, and *Neisseria (Proteobacteria)* (Charlson et al., 2011; Yang et al., 2018; Zhu and Chang, 2023). In addition to bacteria, fungi and viruses also contribute to the lung’s microbial environment. A study on post-antibiotic mouse colonies suggests that the presence of fungi can influence the composition of lung bacteria and the host’s response (Erb Downward et al., 2013). There is relatively less reporting on viruses in the microbiota, but viruses are also present in the blood of healthy individuals. Viruses exhibit high specificity to their hosts and are relatively stable (Minot et al., 2011; Abeles et al., 2014). Under normal circumstances, the lung microbiota is in a balanced state of migration and elimination, with various symbiotic microorganisms in relatively balanced and stable quantities (Spijkerman et al., 2012; Mika et al., 2015). When the lung microbiota is imbalanced, it can affect the immune microenvironment by releasing metabolic products, inducing inflammatory processes, producing bacterial toxins that alter the stability of the host’s genome, and increasing levels of carcinogenic microbial metabolites, thereby leading to the occurrence and development of diseases (**Table 1**) (Mao et al., 2018; Tsay et al., 2021; Campbell et al., 2022). For example, environmental exposures such as cigarette smoke, PM2.5 and air pollutants can lead to increased respiratory symptoms and lung damage. Elevated levels of *Atopobium*, *Actinomyces* and *Prevotella* have been reported in smokers compared to non-smokers). In addition, fungal taxa and especially *Cladosporium* are associated with PM2.5 concentrations (Lin et al., 2023). Exposure to PM2.5 can lead to lung inflammation and oxidative stress (Wang S. M. et al., 2022).

**Table 1:** Summary of key findings on the lung microbiota in chronic pulmonary diseases.

Disease	Sample size	Key finding	Reference
COPD	sputum	The abundance of *Streptococcus, Staphylococcus, Prevotella and Gemella* increases	(Opron et al., 2021; Yi et al., 2022)
COPD	sputum	Airway microbiome-derived IAA mitigates neutrophilic inflammation, apoptosis, emphysema and lung function decline, via macrophage-epithelial cell cross-talk mediated by interleukin-22.	(Yan et al., 2022)
Bronchial asthma	–	*Rhinoviruses* are the most common pathogen that triggers asthma	(Jackson and Gern, 2022)
Bronchial asthma	nasal secretion	*Staphylococcus* is associated with alleviation of asthma symptoms, while *Moraxella* is associated with exacerbation of asthma	(McCauley et al., 2019)
IPF	Lung/bulf	*Epstein-Barr virus* (EBV), *Cytomegalovirus* (CMV), *Human Herpesvirus 7* (HHV-7) and *Human herpesvirus 8* (HHV-8) were associated with a significant elevation in the risk of IPF	(Sheng et al., 2020)
IPF	Lung (mouse)	*Actinomyces* and *Prevotella* promote pulmonary fibrosis in mice through IL-17B signaling	(Yang et al., 2019).
Lung cancer	Airway brushings	*Streptococcus* and *Veillonella* promote lung cancer through the upregulation of the ERK and PI3K signaling pathways	(Natalini et al., 2023a; Tsay et al., 2018)
Lung cancer	Lung (mouse/ human)	*Aspergillus sydowii* promotes tumor progression by inhibiting cytotoxic T lymphocyte activity and PD-1^+^ CD81^+^ Tcell aggregation	(Liu et al., 2023)

### 2.2 Chronic obstructive pulmonary disease

Chronic obstructive pulmonary disease (COPD) is defined by persistent inflammation in the airways, parenchymal part of lung tissue, and pulmonary vessels, and it is progressive and irreversible, making it one of the major contributor of death from chronic lung diseases (Devadoss et al., 2019). COPD has several risk factors, including smoking, genetic factors, environmental pollution, and infection (Labaki and Rosenberg, 2020). Colonization and infection of airway bacteria are the main triggering factors for acute exacerbations of COPD (Meldrum et al., 2024). On one hand, bacteria release bacterial products such as oligosaccharide lipids or other soluble bacterial toxins, causing damage to airway epithelial cells. On the other hand, it can cause local inflammatory reactions, with inflammatory cells releasing cytokines and increasing elastase activity, disrupting the balance of elastase/anti-elastase systems, thereby promoting the progression of COPD and leading to irreversible lung damage (Leung et al., 2017; Pathak et al., 2020; Isaacs et al., 2023). Compared to healthy individuals, the relative abundance of *Actinobacteria* decreases in COPD patients, while the relative abundance of *Haemophil*us increases, which shows a positive correlation with interleukin-8 (IL-8) levels in sputum (Wang Z. et al., 2021). Another study indicates that the presence of a microbial community in sputum dominated by *Proteobacteria* in COPD patients is associated with poorer lung function and disease progression (Dicker et al., 2021). Furthermore, clinical phenotypes of COPD can be distinguished by respiratory microbiota and can better predict patient response to antibiotic therapy (Wang Z. et al., 2021). For example, patients with a neutrophilic inflammatory phenotype are often accompanied by bacterial infections and require antibiotic treatment, while patients with an eosinophilic inflammatory phenotype often show no signs of infection. The alpha diversity of the lower respiratory tract microbiota decreases in COPD patients after glucocorticoid treatment, with an increase in *Moraxella* and *Haemophilus* abundance and a decrease in *Streptococcus* abundance, while the use of antibiotics shows opposite results (Wang et al., 2016). The respiratory microbiota undergoes significant changes during acute exacerbations of COPD compared to stable periods, with decreased microbial diversity and increased abundance of *Proteobacteria*, particularly *Haemophilus* and *Moraxella*, and a significant decrease in *Staphylococcus* (Sun et al., 2020; Zheng et al., 2022). Viral infections are among the factors contributing to acute exacerbations, and nasal virus infection in COPD patients can enhance neutrophil elastase-mediated antimicrobial peptide degradation. This virus-induced increase in secondary bacterial infections leads to an increase in *Haemophilus* abundance and microbial dysbiosis in the lungs (Zheng et al., 2022).

Respiratory viruses and fungi are associated with exacerbation of COPD. *Rhinovirus* is the most common type of viral infection that exacerbates COPD (Stolz et al., 2019). In addition, *Influenza Virus* and *Respiratory Syncytial Virus* (RSV) are frequently detected in the respiratory tract of COPD patients (Simon et al., 2023). COPD patients have high expression of ACE2 (the receptor for SARS-CoV-2), making them more susceptible to COVID-19 (Higham et al., 2020). Multiple studies have shown that virus-induced COPD exacerbations may be related to interferon IFN (Garcia-Valero et al., 2019; Collinson et al., 2021). In addition to viruses, the role of fungi in COPD patients is gradually being recognized, and fungal sensitization is prevalent in COPD patients and associated with poor outcomes (Tiew et al., 2020). For example, a prospective multicenter study from Singapore found that *Aspergillus* sensitization is associated with COPD exacerbations (Tiew et al., 2023).

It has been reported that most COPD exacerbations are caused by bacterial or viral infections (Kim et al., 2024). Due to the downregulation of pattern recognition receptors (PRRs) such as Toll-like receptors (TLRs) and Nod-like receptors (NLRs) on the airway epithelial cells of COPD patients, the recognition ability of bacterial pathogens is impaired, leading to delayed and insufficient immune responses (Sidletskaya et al., 2020). Bacterial infections in COPD patients can induce oxidative stress, produce reactive oxygen species (ROS), and impaired phagocytic function (Yamasaki and van Eeden, 2018; Singh et al., 2021). Most respiratory viruses target airway epithelial cells, causing epithelial barrier disruption, microvascular dilatation, edema, and immune cell infiltration, which can lead to increased levels of CD8^+^ T cells, neutrophils, eosinophils, TNF-n and IFN-n in COPD patients (Paats et al., 2012). Additionally, COPD is characterized by specific fungal genera such as *Aspergillus*, *Curvularia* and *Penicillium* (Tiew et al., 2021). Environmental exposure is the main source of fungal allergens and *Aspergillus* can form biofilms on the airway epithelial cells of COPD patients to resist host immunity and antifungal therapy. It can also lead to impairment of neutrophil function and increased apoptosis, resulting in disease progression (Tiew et al., 2020).

### 2.3 Bronchial asthma

Bronchial asthma (asthma) is a common respiratory system disease characterized by recurrent wheezing, shortness of breath, chest tightness, or coughing. Colonization or infection of microorganisms in the upper respiratory tract and lower respiratory tract can lead to the onset of asthma (Gon and Hashimoto, 2018). The mechanism involved mainly includes promoting IgE synthesis and histamine release, leading to a hypersensitive state of the body, promoting the release of various cytokines. This process triggers numerous allergic responses, such as eosinophilic inflammation, transformation of immunoglobulin (IgG) into IgE, promotion of B cell proliferation, goblet cell transformation, and the consequent mucus secretion, exacerbating airway inflammation and damage, leading to airway spasm, edema, and exudation. Changes in the local microbial community lead to local immune dysfunction, resulting in the occurrence of asthma (Whetstone et al., 2022). Studies have shown that changes in the microbiota in asthma have a significant impact on the pathophysiology of the disease (Barcik et al., 2020; Santos et al., 2021). For example, the abundance of neutrophils in the sputum of asthma patients is related to the levels of specific taxa, including *Moraxella* (Ma et al., 2021). Researchers have detected a variety of microorganisms in the lower respiratory tract microbiota of asthmatic children, including *Bacteroides*, *Faecalibacterium*, *Roseburia*, *Moraxella*, *Staphylococcus*, and *Streptococcus* (Goldman et al., 2018; Al Bataineh et al., 2020). Among these microorganisms, *Staphylococcus* is associated with alleviation of asthma symptoms, while *Moraxella* is associated with exacerbation of asthma (McCauley et al., 2019). In addition, an increase in *Proteobacteria* and an elevation of non-*Proteobacteria* such as *Pseudomonas*, *Clostridium*, and members of the family *Enterobacteriaceae* have been observed in the airways of asthma patients (Azim et al., 2021), and are significantly associated with the expression of Th17-related genes, which may lead to recruitment of neutrophils (Wilburn et al., 2023). *Rhinovirus* is the most common pathogen triggering asthma, followed by *Human Bocavirus* and *Human Metapneumovirus* (10-25% positivity) (Coverstone et al., 2019). CDHR3 has been found to be highly expressed in differentiated bronchial epithelial cells and acts as a receptor for Rhinovirus C to increase the risk of respiratory disease (Bonnelykke et al., 2018). These studies indicate that changes in the microbial community are not only related to asthma but may also play a role in the changes of asthma symptoms.

### 2.4 Idiopathic pulmonary fibrosis

Idiopathic Pulmonary Fibrosis (IPF) is the most common and prevalent type of pulmonary fibrosis. Activation of cells in the alveolar region leads to the release of a large number of cytokines and growth factors, promoting the recruitment, proliferation and differentiation of lung fibroblasts into myofibroblasts, resulting in progressive lung parenchymal damage. This process leads to irreversible decline in lung function and even respiratory failure (Moss et al., 2022). One multicenter study showed that *Human Herpesvirus 7* (HHV-7), *Human Herpesvirus 8* (HHV-8), *Epstein-Barr virus* (EBV), and *Cytomegalovirus* (CMV) were associated with a significantly increased risk of IPF (Sheng et al., 2020). Testing of the lower airways of IPF patients has revealed an increased abundance of *Haemophilus*, *Veillonella*, *Streptococcus*, and *Neisseria* (Zhang T. et al., 2023). It has been reported a positive correlation between the concentration of IL-6 in the alveoli of IPF patients and the relative abundance of *Firmicutes*, while the concentration of IL-12p70 in the alveoli was negatively correlated with the relative abundance of *Proteobacteria* (O'Dwyer et al., 2019). Researchers have found that peptides secreted by *Staphylococcus* induce apoptosis of lung epithelial cells and collagen deposition, leading to acute exacerbation of IPF and further inhibition of these apoptotic peptides can improve acute exacerbation of pulmonary fibrosis (D'Alessandro-Gabazza et al., 2020). Another study demonstrated that *Actinomyces* and *Prevotella* promote pulmonary fibrosis in mice through IL-17B signaling (Yang et al., 2019). In a mouse model of bleomycin-induced pulmonary fibrosis, germ-free mice have a higher mortality rate compared to conventional mice, demonstrating the complex relationship between lung microbiota changes and IPF-related inflammatory activity. In conclusion, we can find that microorganisms may promote or inhibit IPF through certain key signaling pathways.

### 2.5 Lung cancer

The incidence and mortality of lung cancer are among the highest globally (Leiter et al., 2023). Approximately 90% of lung cancer cases are attributed to risk factors such as smoking, tobacco smoke, air pollution and other carcinogens (Qi et al., 2023; Xue et al., 2023). Lung cancer patients show decreased alpha diversity and altered bacterial composition. Researchers have found a transition in dominant bacterial taxa from *Firmicute*s to *Bacteroidetes* in saliva and bronchoalveolar lavage samples of lung cancer patients (Xie et al., 2022) and a correlation between intratumoral bacteria and tumor type and subtype, patient smoking status and immune therapy response (Nejman et al., 2020). Chronic airway inflammation can increase susceptibility to lung cancer, suggesting that airway dysbiosis may be one of its pathogenic mechanisms (Goto, 2022). Studies have found enrichment of the airway commensal bacteria *Megasphaera* and *Veillonella* in the bronchoalveolar lavage fluid (BALF) of lung adenocarcinoma patients (Guo et al., 2022). Other researchers have found an abundance of *Streptococcus* and *Veillonella* in the lower respiratory tract of lung cancer patients, leading to upregulation of the ERK and PI3K signaling pathways, promoting lung cancer cell proliferation (Tsay et al., 2018). Bacterial metabolites such as reactive oxygen and nitrogen species can directly cause DNA damage and disrupt multiple signaling pathways, creating a pro-carcinogenic environment (Dong et al., 2021; Ho et al., 2021). In addition to bacteria, fungi such as *Blastomyces* (Li and Saxena, 2022) and *Aspergillus sydowii* (*A. Sydowii*) (Liu et al., 2023) have also been found in lung tumor tissues. A recent study revealed that the intratumoral fungus *Aspergillus sydowii* promoted lung cancer progression through IL-1ughsionlTE expansion and activation of myeloid-derived suppressor cells (MDSCs), and the enrichment of *Aspergillus* was closely associated with poorer prognosis in lung cancer patients (Liu et al., 2023). The relationship between microorganisms and their microenvironment with tumors is very close and a more comprehensive understanding of the character of the microbiota in lung cancer is essential. Given that the lung microbiome is associated with the prognosis of lung cancer patients and can promote lung cancer progression through key signaling pathways, it can serve as a critical diagnostic and preventive biomarker for lung cancer staging, genotyping and risk stratification (Poore et al., 2020).

## 3 Mechanism of microorganisms and host interactions

Some studies have shown that microorganisms interact with the host through metabolites to regulate signaling pathways. For example, the indole IAA produced by *Lactobacillus* alleviates neutrophil inflammation, cell apoptosis, emphysema and lung function decline through IL-22-mediated macrophage-epithelial cell interaction (Yan et al., 2022). Additionally, *Lactobacillus* can metabolize dietary tryptophan into indole, thereby inhibiting tumor immunity and promoting the growth of pancreatic ductal adenocarcinoma (Hezaveh et al., 2022). Given that host-microbiota interactions are bidirectional, microbial-derived metabolites may interact with host macromolecules and affect their responses. Short-chain fatty acids (SCFAs), such as acetate, propionate and butyrate, reduce tumor necrosis factor TNF-o production by inhibiting histone deacetylase (HDAC) and suppressing the transcription factor NF-to (Chambers et al., 2018). Metabolomic changes can predict asthma outcomes, as researchers have found a positive correlation between 5’-AMP, uracil and niacinamide with asthma exacerbations using non-targeted sputum metabolomics (Liu et al., 2022). A study revealed that metabolites related to lipid peroxidation in urine samples are linked to the severity of asthma, lung function and eosinophilic inflammation in non-obese asthmatic individuals (Wang C. et al., 2021). Additionally, in a mouse model, fecal microbiota from patients with COPD was demonstrated to play a role in the onset of COPD (Li et al., 2021) and COPD patients and healthy individuals exhibit distinct microbial and metabolic features in fecal samples (Bowerman et al., 2020). NMR analysis of urine from pneumonia patients indicates that specific metabolic profiles can be used to differentiate pneumococcal pneumonia from pneumonia caused by other bacterial strains (Green et al., 2023). NMR analysis of urine from pneumonia animals infected with either *Streptococcus* pneumoniae or methicillin-resistant *Staphylococcus aureus* (MRSA) revealed different metabolic profiles (Green et al., 2023). These results suggest that metabolomics has potential in the diagnosis and monitoring of pneumonia.

## 4 The connection between lung microbiota and gut microbiota

All parts of the human body are colonized by microorganisms, with the gut harboring the highest density. The gut microbiota, comprising tens of trillions of symbiotic bacteria, fungi, archaea and viruses (Zhou et al., 2021), has garnered significant attention. The emerging concept of the gut-lung axis underscores the intricate interplay between lung and gut microbiota (Anand and Mande, 2018). Clinical studies indicate that lung diseases may be associated with gut microbiota (Li et al., 2021) and alterations in lung microbiota can lead to changes in the composition and metabolism of gut microbiota (Bowerman et al., 2020). Conversely, translocated gut microbiota and their products can influence pulmonary immunity (Özçam and Lynch, 2024). For instance, fecal microbiota from COPD patients has been shown to contribute to COPD development, with gut microbial-derived lipopolysaccharides (LPS) exacerbating COPD progression in mice (Li et al., 2021). Bowerman et al. identified a disease-related network linking *Streptococcus parasanguinis_B* with COPD-associated metabolites, such as N-acetylglutamate and its analogue, providing valuable insights for COPD biomarker discovery (Bowerman et al., 2020). Additionally, fecal microbiota transplantation in healthy mice has been demonstrated to attenuate emphysema development by inhibiting inflammation both locally and systemically, and by altering gut microbiota composition (Jang et al., 2020).

## 5 Pulmonary microbiome testing tools

### 5.1 Amplicon sequencing

Currently, amplicon sequencing and metagenomics Next-Generation sequencing (mNGS), as well as targeted sequencing (tNGS), are the predominant sequencing technology used in microbiome research (Han et al., 2022). Amplicon sequencing targeting 16S rRNA region of bacteria gene or ITS region of fungi (Gao et al, 2023). Primarily involves PCR amplification of a partial region followed by high-throughput sequencing to detect sequence variations and abundance information. When studying lung microbes, the V4 or V3V4 region sequence is most often selected. For fungi, often a portion of ITS1 or ITS2 sequence was characterized. This method reveals the types, relative abundance, and evolutionary relationships of microorganisms in environmental samples (Liu et al., 2021). Due to its high specificity, sensitivity, throughput and simple data analysis process, amplicon sequencing is favored by many researchers and remains irreplaceable in microbiota studies (Zhang W. et al., 2023). However, this method has limitations as it is not applicable for sequencing viral genomes due to the lack of a conserved gene similar to the 16S rRNA gene in these organisms. Additionally, limited universal primers, methodological constraints, and high host contamination restrict the accurate reflection of actual microbial community structures in samples (**Table 2**) (Weinroth et al., 2022).

**Table 2:** Comparison of microbial sequencing method.

	Advantages	Disadvantages	Applications
16s rRNA	High abundance; Low cost; No host contamination	Unable to detect fungi and viruses; Low resolution	Bacterial Identification (Liu et al., 2023)
ITS	Low cost; High sensitivity	The selection of primers affects the sequencing results	Fungi identification (Yi et al., 2022)
mNGS	High resolution; No probe synthesis; Detect unknown species	High DNA quality requirement ; Host contamination ; Expensive	Detection of bacteria, fungi and viruses (Han et al., 2023; Li et al., 2024)

### 5.2 Metagenomics next-generation sequencing

In the clinical setting, mNGS is applied to infectious disease diagnosis, respiratory microbiome analysis, human host response analysis to infections, drug resistance prediction, colonization and infection differentiation, as well as the identification of tumor-related viruses and their genomic integration sites in various syndromes and sample types (Diao et al., 2022; Ibañez-Lligoña et al., 2023). The main advantage of mNGS is its unbiased sampling, as it maps the obtained sequence information to microbial resource databases, overcoming the limitations of targeted detection methods by characterizing all microorganisms in the human body system, including viruses, fungi, bacteria and parasites, in a single test (Qin et al., 2022). mNGS can provide a comprehensive view of the microbial community structure and function. However, mNGS also has limitations, as most bacteria can only be identified at the genus level, and contamination may occur during sampling or DNA extraction processes (Wensel et al., 2022).

### 5.3 Targeted next-generation sequencing

Targeted sequencing (tNGS), also known as pathogen-targeted sequencing, detects known pathogenic microbial information in samples through multiplex PCR. tNGS specifically amplifies target genes of interest, thereby avoiding the influence of the host and sampling process, making it a highly sensitive and cost-effective optimization method (Sibandze et al., 2022). tNGS technology is used for the detection of known pathogens and drug-resistant genes (Wang S. et al., 2022; Yi et al., 2024)]. However, this technology faces challenges in its development and application, including low detection rates due to non-specific amplification, primer dimer interference, and amplification bias, as well as the need for improvement in the recognition and detection of new pathogens and rare specimen capabilities (Li et al., 2021).

### 5.4 Metatranscriptomics

Metatranscriptomics can assess the gene expression of microorganisms to explain their composition and function in the environment, such as the lungs, oral cavity and gut. It allows us to understand the interactions between the microbe and the host (Ren et al., 2019). It overcomes the limitations of polymerase chain reaction (PCR) amplification and is not limited to the analysis of specific bacteria, making it particularly advantageous in characterizing host-microbe gene expression. However, it also has its limitations, such as the possibility of host RNA contamination and RNA degradation during processing (Gao et al., 2023). Therefore, it requires extra caution. In the analysis process, even using the same database, different macro-genomic analysis methods can sometimes produce different results.

### 5.5 Microbial metabolomics

Microbial metabolomics can provide accurate information about the actual physiological status of microorganisms, identifying immunomodulatory metabolites to reflect the health status of the environment or evidence of ecological imbalance (Liu et al., 2022). NMR, GC-MS, and LC-MS are several commonly used tools in microbial metabolomics (Ye et al., 2022). Sample pretreatment techniques based on NMR metabolomics are relatively simple, allowing for objective and non-destructive sample evaluation and identification, with stable and strong repeatability ofdetection results (Traverso et al., 2018; Valentino et al., 2020). NMR is better suited for analyzing compounds that are difficult to ionize and require derivatization. However, due to limited sensitivity, MS is a better choice for achieving higher sensitivity and separation efficiency. LC-MS and GC-MS can detect thousands of different metabolites in various metabolic areas at the micromolar to millimolar leve l (Misra, 2020). Currently, mass spectrometry ismainly used in clinical microbiological identification (Bauermeister et al., 2022). LC-MS is primarily used for the analysis and detection of stable compounds and large molecular compounds (including proteins, peptides, and polymers). Compared to LC-MS, GC-MS can relatively easily identify a larger proportion of metabolites, as well as separate, identify, and quantify molecules in mixed samples, making it the preferred tool for the analysis of volatile small molecule metabolites (Weisskopf et al., 2021).

To thoroughly understand the role of microorganisms in the human body and their impact on human health, it is necessary to use a combination of omics tools. Multi-omics integration is an inevitable trend in future research, and it is hoped that through multi-omics, we can gain a deeper understanding of the role of lung microorganisms in respiratory diseases and develop more effective disease treatment strategies.

## 6 Conclusion

Existing research indicates that the human microbiome plays a crucial role in the development and progression of human diseases, with the changes in the microbiome and its metabolites having a significant impact on the pathophysiology of diseases. Therefore, in order to conduct a more comprehensive study of the microbiome, we have listed several of the most mainstream microbiome sequencing methods. These sequencing tools can help us identify the presence of microorganisms, understand the dynamic changes of microbiota in diseases, assess their functions and their direct impact on the host. Furthermore, combined with metabolomics and other multi-omics methods for joint analysis, they can deepen our understanding of the molecular mechanisms underlying microbiome-related diseases.

## 7 Discussion

Despite some achievements in certain aspects, the lung microbiome still faces challenges. Sampling is a key aspect in lung microbiome research. Compared to the skin and gut, the biomass in the lungs is low, making sampling and detection difficult (Sulaiman et al., 2021). Additionally, the upper respiratory tract serves as the entry point connecting with the external environment and is typically the first point of contact for inhaled pollutants and pathogens, thus sample data from the upper respiratory tract may influence disease prognosis (Kumpitsch et al., 2019; Tiotiu et al., 2020). Another significant limitation is that existing metabolomic analysis techniques cannot distinguish whether certain metabolites, such as histamine, originate from the host or from microorganisms if both produce the same metabolite (Yamauchi and Ogasawara, 2019; Krell et al., 2021). Furthermore, to better understand the relationship between the role of the respiratory microbiome and disease progression, more longitudinal studies are crucial.

In the clinical medical field, microbiota transplantation has been applied clinically, but only in the gastrointestinal tract (Waller et al., 2022). The impact of altering the respiratory microbiota on clinical treatment is unknown, but it will be a direction for future research. With the continuous advancement of technology, high-throughput sequencing techniques will continue to have a key role in microbiome research. Recently, two innovative technologies, 2bRAD-M simplified metagenome sequencing and MobiMicrobe high-throughput single-cell genome sequencing, have emerged (Sun et al., 2022; Zheng et al., 2022). These technologies can effectively handle low biomass, severe degradation, and high host-contaminated samples, overcoming the limitations of mainstream technologies and offering unique core advantages. In future research, these technologies will demonstrate their strengths and value in development and application, enabling tailored microbial intervention strategies for different individuals and applying them to the prevention and treatment of clinical diseases. This will give us a comprehensive understanding of the microbial communities in the human body.
